# Network of spin-based sensors constrains topological dark matter

**DOI:** 10.1093/nsr/nwag492

**Published:** 2026-08-14

**Authors:** Dmitry Budker

**Affiliations:** Department of Physics, Mathematics, and Computer Science, Johannes Gutenberg University, Germany; Department of Physics, University of California, USA

Imagine you come into possession of several of the most advanced and sophisticated sensors capable of detecting the minutest variations in magnetic fields. What will you do with them?

**Figure 1.**
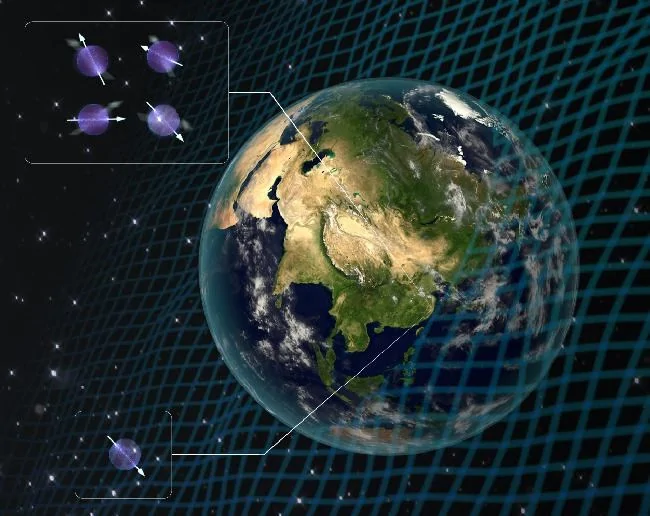
A network of spin-based sensors located in two cities in China: Hefei (four sensors) and Hangzhou (one sensor). The sensors are synchronized using the global-positioning-system timing. The network is designed to search for encounters with so-called topological dark matter depicted as a gridded surface traversing the Earth. Image courtesy of Dr. Yuanhong Wang.

A team of researchers from several leading research laboratories in China and a collaborator from the Jagiellonian University in Krakow, Poland, have done something non-obvious [1]: they placed their spin-based sensors [2] within advanced multilayer magnetic shields that screened the sensors from the effects of magnetic fields. Moreover, they have operated the sensors as a network that is time-synchronized using the global positioning system (GPS), with several sensors located in Hefei, and one sensor at Hangzhou, over 300 km away (Fig. 1).

The explanation for such an uncommon sensor configuration is the specific goal of the experiment. Even though the elements of the detectors (atomic vapor cells, diode lasers, photodiodes, …) are typical for a laboratory experiment in atomic, molecular and optical (AMO) physics, the network is, in fact, an astrophysical observatory, a type of haloscope searching for dark matter that is believed to be present in our and other galaxies in the form of galactic halos, hence the term for such detectors.

Dark matter is nearly a century-old problem. Various astrophysical observations suggest that *≈*80% of matter in the universe is in the form of dark matter—a substance that seems to only weakly interact with both normal matter and itself (thus penetrating the magnetic shielding), however, fully participating in the gravitational interactions. One of the manifestations of the apparent presence of dark matter in a galaxy is that the stars at the galactic periphery move much too fast compared to what one would expect given the amount of normal matter in the galaxy that we observe. From these observations, one infers the average local density of the galactic dark matter of *≈**0.4 *GeV/${\rm c}^2$ cm$^{-3}$ (where *\rm c* is the speed of light), or roughly a proton-mass equivalent per 3 cm$^{3}$.

Interestingly, despite the strong evidence for the presence of dark matter, its origin and composition are still unknown, and many diverse ideas have been put forward, motivating a broad net of searches, each tailored for a specific type of hypothetical dark matter. A particular scenario explored in Wang *et al.* [1] is that of ultralight bosonic dark matter (UBDM), which manifests as a pseudomagnetic field that affects nuclear spins but not the electrons in the magnetic shield. As a result, the pseudomagnetic field from dark matter penetrates the magnetic shield without significant attenuation, while the noisy ambient magnetic field is nearly completely canceled.

Networks of shielded magnetometers were proposed over a decade ago [3] to search for so-called topological UBDM that, rather than being more or less homogeneously distributed in our galactic neighborhood, comes in the form of zero-dimensional clumps, one-dimensional strings or, as originally discussed, two-dimensional domain walls. If such a wall crosses Earth, it would cause a particular temporal pattern of sensor responses, from which, knowing the geographical location of the sensors and relying on the GPS timing synchronization, one can, in principle, infer the direction and speed of the domain wall.

There are other networks looking for topological dark matter, e.g. the Global Network of Optical Magnetometers for Exotic physics searches (GNOME) [4], including sensors in China; however, the newly reported experiment has searched a previously unexplored parameter space at a level of sensitivity improving on (model-dependent) astrophysical constraints. Specifically, the experiment was sensitive to the dark-matter particles with rest energies in the range of 10$^{-11}$—$2\times 10^{-7}$ eV, reaching out to interaction strength $1/f_{{\rm int}}$ with the interaction-strength parameter as high as $f_{{\rm int}}\approx 4\times 10^{10}$ GeV. The achievements of the new experiment are largely due to an innovative data-analysis technique optimized for extracting the sought-for signal from noise.

These results tighten the constraints on topological dark matter, leaving fewer hiding places for these elusive cosmic objects!
